# Supplementary figures and images for: Treatment Outcomes in Spinal Tumors According to Patients’ Perspectives: A Focus on Indeterminate Spinal Instability
Source: Curr Oncol. 2025 Jan 13;32(1):38. doi: 10.3390/curroncol32010038 (PMC11763783; doi:10.3390/curroncol32010038)

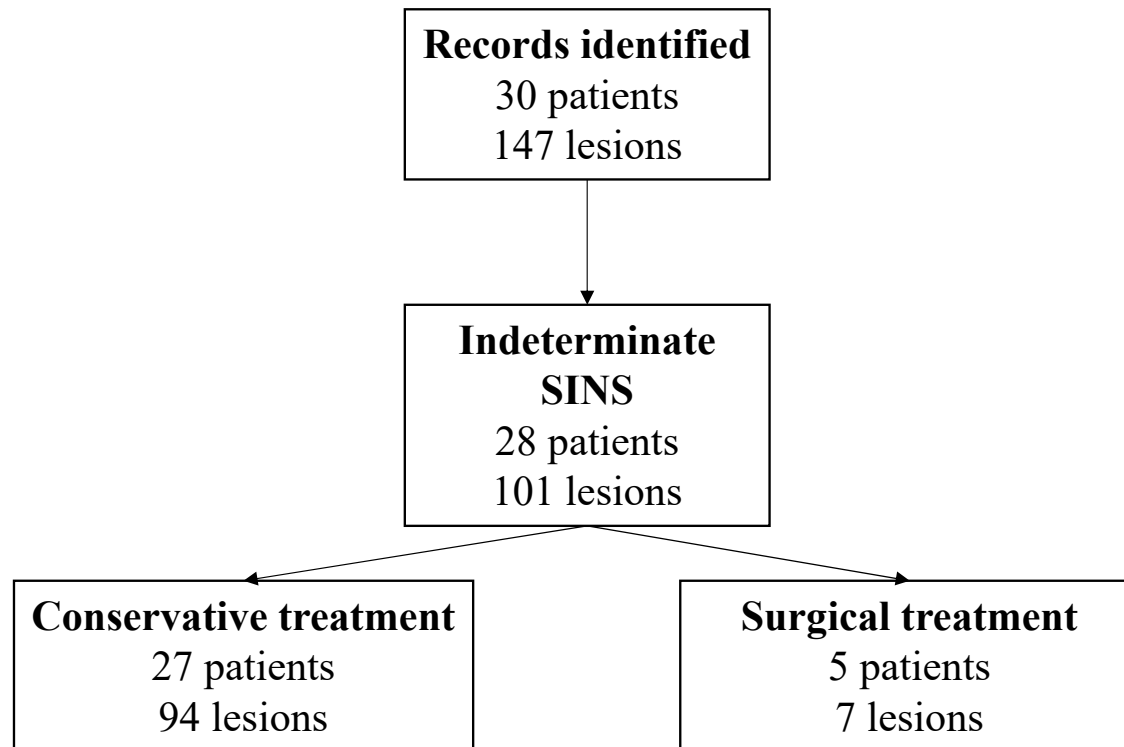

**Figure S1.** Flow chart Patient Selection.

Supplement: Supplementary file 1 [file curroncol-32-00038-s001.zip › curroncol-3390654-supplementary.pdf]
